# Can Simulator Sickness Be Avoided? A Review on Temporal Aspects of Simulator Sickness

**DOI:** 10.3389/fpsyg.2018.02132

**Published:** 2018-11-06

**Authors:** Natalia Dużmańska, Paweł Strojny, Agnieszka Strojny

**Affiliations:** ^1^R&D Unit, Nano Games sp. z o.o., Kraków, Poland; ^2^Institute of Applied Psychology, Faculty of Management and Social Communication, Jagiellonian University, Kraków, Poland

**Keywords:** simulator sickness, temporal aspects, time, virtual reality, VR

## Abstract

Simulator sickness is a syndrome similar to motion sickness, often experienced during simulator or another virtual reality (VR) exposure. Many theories have been developed or adapted from the motion sickness studies, in order to explain the existence of the syndrome. The simulator sickness can be measured using both subjective and objective methods. The most popular self-report method is the Simulator Sickness Questionnaire. Attempts have also been made to discover a physiological indicator of the described syndrome, but no definite conclusion has been reached on this issue. In the present paper, three temporal aspects of the simulator sickness are discussed: the temporal trajectory of the progression of simulator sickness, possibility of adapting VR users in advance and persistence of the symptoms after VR exposure. Evidence found in 39 articles is widely described. As for the first aspect, it is clear that in most cases severity of the simulator sickness symptoms increases with time of exposure, although it is impossible to develop a single, universal pattern for this effect. It has also been proved, that in some cases a threshold level or time point exists, after which the symptoms stop increasing or begin to decrease. The adaptation effect was proved in most of the reviewed studies and observed in different study designs – e.g., with a couple of VR exposures on separate days or on 1 day and with a single, prolonged VR exposure. As for the persistence of the simulator sickness symptoms after leaving the VR, on the whole the study results suggest that such an effect exists, but it varies strongly between individual studies – the symptoms may persist for a short period of time (10 min) or a relatively long one (even 4 h). Considering the conclusions reached in the paper, it is important to bear in mind that the virtual reality technology still evokes unpleasant sensations in its users and that these sensations should be cautiously controlled while developing new VR tools. Certainly, more research on this topic is necessary.

## Introduction

### Virtual Reality – A Definition and the Most Commonly Used Devices

The simplest definition of virtual reality states that is “the use of computer-generated virtual environments and the associated hardware to provide the user with the illusion of physical presence within that environment” (Jayaram et al., 1997, p. 576). Virtual reality systems are widely used in the fields of scientific research (e.g., Anderson-Hanley et al., 2011), anxiety disorders therapy (e.g., Gerardi et al., 2010; Łukowska, 2011) or for professional training [e.g., in the army – (Braithwaite and Braithwaite, 1990); fire department – (Bliss et al., 1997); aviation – (Kennedy et al., 2000); medicine – (Bric et al., 2016)].

Many different virtual reality hardware systems and devices have been developed over the years and will be briefly described herein. Nowadays, the most popular are the head-mounted devices (HMDs), such as HTC Vive or Oculus Rift. The VR user wears a headset and holds two controllers which enable them to move and interact in a three-dimensional environment. Such devices are now being sold commercially. According to a recent Business Insider report (Hollander, 2018), there are four main VR headset types: stand-alone (which do not need any additional hardware to function), smartphone-powered, PC-powered and game console-powered. The report predicts that the stand-alone headsets will grow in popularity in the coming years. This could be of advantage for research employing the VR technology, as eliminating the wire which connects the headset to a PC or a console will make conducting experiments with multiple participants at the same time much easier.

Another example of a VR system is a CAVE (cave automatic virtual environment). In such system, the environment is displayed and generated on several projectors, directed to the walls of the room and the user wears 3D glasses.

Different additional devices are used in order to provide the VR user with a realistic, multisensory experience. For example, treadmills are often used to simulate movement in the virtual environment (e.g., Jaeger and Mourant, 2001; Sinitski et al., 2018). For driving and flight simulators, a part of a plane cockpit or a body of a car may be used (e.g., Feenstra et al., 2011; Domeyer et al., 2013; Reinhard et al., 2017).

### Definition of Simulator Sickness

Simulator sickness is a syndrome similar to motion sickness and can be experienced as a side effect during and after exposure to different virtual reality environments. Originally, the term “simulator sickness” was linked to effects induced by simulators consisting of a platform, often mobile, and with the visual stimuli generated by a computer, without head-tracking. The invention of HMDs led to developing another term, “cybersickness,” as such devices generate another issues, which may also lead to the unpleasant symptoms, such as the delay between actual head movements and the generated image. However, nowadays both of the terms are being used by researchers to describe the unpleasant symptoms evoked by the virtual reality technology (e.g., Sharples et al., 2008; Bruck and Watters, 2011; Serge and Moss, 2015; Lee et al., 2017).

The symptomatology and severity of the malaise depend on many variables – e.g., age, gender, stress, anxiety, one’s individual proneness to such ailment or the characteristics of the simulator itself (Kolasinski, 1995; Cobb et al., 1999; Mourant and Thattacherry, 2000; Jaeger and Mourant, 2001; Lin et al., 2002; Sharples et al., 2008; Brooks et al., 2010; Bruck and Watters, 2011; Classen et al., 2011; Moss and Muth, 2011; Zużewicz et al., 2011; Biernacki and Dziuda, 2012; Dziuda et al., 2014; Helland et al., 2016; Lee et al., 2017). Lin et al. (2002) have also suggested that a relationship between one’s enjoyment experienced during simulator training may lead to alleviation of the simulator sickness symptoms. A very detailed list of variables, which may have influence on simulator sickness occurrence and severity, may be found in the report by Kolasinski (1995).

The main aims of this paper are to summarize the existing knowledge on simulator sickness with emphasis on its temporal aspects, to provide an overview of research on this topic and to propose further research directions and practical implications for virtual reality developers.

Firstly, the most common theories which could serve as an explanation of the simulator sickness phenomenon will be discussed. Secondly, the methods of simulator sickness measurement, both subjective and objective, will be described in detail. Thirdly, three temporal aspects of simulator sickness will be discussed based on evidence found in empirical studies. And lastly, general conclusions drawn from the reviewed studies and practical implications for further research will be provided.

### Theories Potentially Explaining Simulator Sickness

Several theories have been developed to explain why individuals suffer from motion sickness. According to authors focused on virtual simulators, they may be also applicable in the field of simulator sickness during exposure to virtual reality (Brooks et al., 2010). The Sensory Conflict Theory, proposed by Reason and Brand (1975), explains motion and simulator sickness through a conflict that arises between different sensory systems; namely the signals from visual, vestibular and non-vestibular proprioceptors differ from one another and inevitably differ with expectations based on previous experience. According to the theory, only the conflict between present sensory information and that retained from immediate past elicits sickness. That is claimed on the basis of observation that continuous exposition to a stimulus results in eventual disappearance of symptoms (adaptation) even if the present conflict still exists (Reason, 1978). The vestibular system, which is responsible for perception and detection of direction, is crucial for occurrence of simulator and motion sickness symptoms (Reason and Brand, 1975).

Reason (1978) proposed the Neural Mismatch Model which identifies the source of simulator sickness in discrepancies between expectations derived on a basis of present moves and contents kept in the neural store which, according to Reason (1978), contains information about typical combination of command signals (efference) and the integrated patterns of inputs from the orientation senses generated by them (reafference). That is the theoretical mechanism of adaptation to motion sickness observed for example by Reason and Brand (1975). To conclude, according to this model, sickness occurs when the received sensory information does not match one’s experiences based on past situations.

Another theory, widely used to explain simulator and motion sickness, is the Postural Instability Theory. Riccio and Stoffregen (1991) have criticized the Sensory Conflict Theory – they state that sensory conflicts such as those described by Reason and Brand (1975) happen very often and are nothing unusual. Furthermore, the difference (or lack of it) between what one’s senses experience and what an individual expects to feel is immeasurable. They have proposed that the symptoms of motion or simulator sickness may be experienced when one has been exposed to long-lasting postural instability and has not yet learned how to adjust to this situation and maintain proper balance. The most vivid example of such phenomenon is the feeling of instability one experiences when traveling by ship. A similar situation occurs during rollercoaster rides as well (Riccio and Stoffregen, 1991).

The two aforementioned theories are most prevalent in the literature concerning simulator sickness. Other theoretical approaches to this phenomenon have been developed as well. The Eye Movement Theory developed by Ebenholtz (1992, 2001), uses the vagus nerve stimulation as an explanation for motion and simulator sickness. The mechanism is initiated by two specific eye movements (namely the optokinetic nystagmus and vestibular ocular response^1^) creating tension in the muscles of the eye, which stimulates the vagus nerve and leads to unpleasant symptoms such as difficulty concentrating, eye strain and headaches.

Bruck and Watters (2011) have also attempted to develop a comprehensive theory of cybersickness. They suggest a following chain of causality: an increase in arousal leads to changes in respiration rate, which causes carbon dioxide levels in cerebral blood flow to decrease. These changes lead to the symptoms of simulator sickness: dizziness, fatigue, difficulty concentrating, fullness of head and anxiety. The authors propose dividing the simulator sickness symptoms into four factors:

(1) general cybersickness factor, including most of the simulator sickness symptoms and indicated by feeling sick (generally) and nausea,
(2) vision factor, including respiration, eyestrain and headache,
(3) arousal factor, including respiration, blurred vision, vertigo, difficulty focusing and concentrating and stomach awareness,
(4) fatigue factor, including the following symptoms: eyestrain, fullness of head, self-reported fatigue, dizziness and blurred vision.

The Evolutionary Theory, proposed by Treisman (1977), originally explains the motion sickness, but its assumptions can be adapted to simulator conditions as well. Treisman (1977) suggests that people experience motion sickness, because – evolutionally – our species has not managed to adapt to new transportation modes yet. Therefore, the human body reacts to sensory conflicts with nausea – it acts as if poison had been ingested (Brooks et al., 2010). It can be assumed that similar reasons may stand behind the simulator sickness symptoms, as the human species had even less time to adapt to the virtual reality conditions. Although this theory does not propose any physiological mechanisms that may be responsible for experiencing simulator sickness, it can give a valuable insight on reasons why such ailment exists.

### Measurements of Simulator Sickness

#### Self-Report Measures

##### Simulator Sickness Questionnaire (SSQ)

Originally published by Kennedy et al. (1993), the Simulator Sickness Questionnaire (SSQ) is a tool widely used for assessing the subjective severity of simulator sickness symptoms. In the pre-experiment part of the questionnaire, information about the current physical condition and participant’s experience with simulators is collected. The questionnaire consists of 16 items, derived from the Pensacola Motion Sickness Questionnaire (MSQ). Data collected during previous simulator studies using the MSQ was gathered and the items describing symptoms with less than 1% frequency of appearance or with no change in frequency between pre- and post-exposure were excluded from further analyses (12 of 28 items of MSQ). The severity of each symptom in the SSQ is measured on a four-point scale (0-3).

According to the results of a factor analysis, the items of the SSQ can be grouped into three factors: nausea (e.g., sweating, difficulty concentrating, stomach awareness), oculomotor disturbance (e.g., headache, eyestrain, blurred vision) and disorientation (e.g., fullness head, dizziness with open and closed eyes, vertigo). The factors are not entirely independent – some of the items were included in more than one factor, e.g., the score on difficulty focusing is used to assess the severity of oculomotor disturbance and disorientation. In total, there are five such items. To calculate scores on each factor, all relevant items’ scores should be added (each factor consists of 7 items) and multiplying the obtained sum by a specific weight: for nausea by 9.54 (therefore the scores on this scale range from 0 to 200.34), for disorientation by 13.92 (scores ranging from 0 to 292.32) and for oculomotor disturbance by 7.58 (with scores ranging from 0 to 159.18).

The overall score can be measured as well and it can serve as an indicator of total severity of the simulator sickness. It is calculated by adding scores on the 16 items and multiplying the achieved sum by 3.74, therefore the total score can range from 0 to 179.52. In addition to the quantitative data, qualitative information about peculiar sensations during the simulator experience and symptoms other that those listed in the main part of the questionnaire can be gathered (Kennedy et al., 1993; Biernacki et al., 2016).

Simulator Sickness Questionnaire has been used in numerous studies (e.g., Lampton et al., 1994; Mourant and Thattacherry, 2000; Jaeger and Mourant, 2001; Lin et al., 2002; Min et al., 2004; Sharples et al., 2008; Bruck and Watters, 2009a,b, 2011; Moss and Muth, 2011; Biernacki and Dziuda, 2014; Brunnström et al., 2017). The brevity and simplicity of the questionnaire are its assets, as in many study designs it is being used at least twice to assess the changes in occurrence and severity of simulator sickness’ symptoms. In most cases SSQ is used as a paper-and-pencil test, but it can also be conducted orally – as in Min et al. (2004) study, where the items of the questionnaire were read to the participants by the experimenter (according to the authors of the study, conducting the SSQ orally requires only circa 30–40 s) or in the study by Moss and Muth (2011), where a cassette was pre-recorded and then played back to the participants.

##### Other self-report measures

It should be noted that in some studies self-report methods of measurement different from the Simulator Sickness Questionnaire had been used – Brooks et al. (2010) report having used the Motion Sickness Assessment Questionnaire, Malińska et al. (2014) used a self-developed, concise questionnaire and Helland et al. (2016) measured subjective severity of simulator sickness symptoms simply by asking – “To what extent did you experience simulator sickness during the driving test?”. Several other authors used other short self-report measures (e.g., McCauley et al., 1990; Helland et al., 2016; Reinhard et al., 2017). As these methods are either a questionnaire originally created for measuring a different ailment or have not been psychometrically tested, they will not be described more widely herein.

#### Physiological Measures

Although a conclusion has not yet been reached on which specific physiological parameters are the best indicators of simulator sickness, some researchers (e.g., Min et al., 2004; Bruck and Watters, 2011; Zużewicz et al., 2011) have tested various physiological variables and some of them appear promising for evaluating simulator sickness without relying on self-report measures or as a supportive method for questionnaires such as SSQ. It has been noted (Min et al., 2004) that during driving (and most of the studies concerning simulator sickness were conducted with various driving simulators) the increase of autonomic nervous system activation may relate to tension, which then causes the heart rate and skin conductance to increase and skin temperature to decrease. Moreover, the physiological measures may be useful, as it has been proved that the subjective evaluation of simulator sickness (e.g., with the SSQ questionnaire) is slightly delayed when compared to the physiological indicators (Min et al., 2004). Therefore, establishing the best physiological indicators of simulator sickness could shed more light on the exact triggering time of the syndrome and therefore allow a more accurate description of the temporal characteristics of simulator sickness.

As no unambiguous physiological indicators of simulator sickness have been discovered, some examples of use of physiological indicators for measuring this syndrome will be described in this paragraph.

##### Autonomic nervous system

###### Respiration (breaths per minute)

According to one of the theories of simulator sickness (or “cybersickness,” as referred to by the authors; Bruck and Watters, 2011), the changes in respiration rate are crucial to evoking the unpleasant symptoms, especially when the person subjected to a virtual reality environment has no control. Respiration loads two factors in the theory of cybersickness developed by Bruck and Watters (2011): Vision and Arousal. They even propose that hyperventilation may be the cause of arousal experienced by individuals exposed to high levels of movement in a virtual reality. Empirical evidence of changes in respiration rate during VR exposure were achieved by Kim et al. (2005) – in their study a decrease in the respiration rate (when compared to baseline levels) was observed. What is more, a positive correlation was observed between respiration rate and the Simulator Sickness Questionnaire scores (for all of the subscales and the total score, with the *r* values oscillating between 0.342 for nausea and 0.392 for the total score).

##### Heart rate

Bruck and Watters (2011) propose that the heart rate may serve as an indicator of simulator sickness, as it had been previously proved that it correlates with such syndrome. In experiments conducted by Cobb et al. (1999) heat rate tended to accelerate during the simulator task and returned to a resting rate in approximately 30 min after completing the task. Furthermore, the heart rate of the participants who reported more severe simulator sickness symptoms was also higher than the heart rate of the individuals who did not experience such unpleasant sensations. Additionally, the heart rate of the participants who showed symptoms of adapting to the VR (virtual reality) conditions during several exposures decreased over the three sessions. Changes in heart rate were observed in a couple of studies. Dahlman et al. (2008, 2009) have noted an increase in heart rate during a VR exposure. In a study by Gavgani et al. (2016) the subjects participated in three roller coaster simulator rides, which took place on separate days. For the first 2 days, an initial tachycardia and tachypnoea that gradually lowered during the ride was observed. No such patterns were discovered on the third day.

###### Other autonomic variables

In the course of research, some other measures of the autonomic nervous system activity have been tested. This paragraph will provide a brief overview of them. Kim et al. (2005) have observed an interesting pattern of the gastric tachyarrhythmia changes – in increased significantly in the first 4 min of virtual reality exposure and then continued to increase until the final 4 min of a 9.5 min trial. The eyeblink rate did also change in the study by Kim et al. (2005) – it decreased in the first minute of the exposure (when compared to the baseline rate), but then increased and in the middle of the trial it was significantly higher than the baseline level. Another interesting measure is the skin temperature – as observed by Kim et al. (2005), when measured at the fingertip, the skin temperature decreased in the middle of the trial and remained significantly lower than the baseline level even after leaving the VR environment. Such decrease in skin temperature was also observed by Chung et al. (2007) and Brooks et al. (2010). Furthermore, according to the results obtained by Kim et al. (2005), the respiratory sinus arrhythmia (a variation in heart rate occurring during breath cycle) increases during VR exposure.

What is interesting about the above mentioned measures is the fact that for all of them, except for skin temperature, positive correlations with the subjective measurement of the simulator sickness (SSQ) were observed (Kim et al., 2005), with the Pearson *r* values ranging between 0.265 (eyeblink rate and oculomotor disturbance scale) and 0.359 (gastric tachyarrhythmia and nausea scale).

Furthermore, in a study by Gavgani et al. (2016), a rapid increase in finger skin conductance levels was observed during the first minute of the VR exposure – the subjects experienced increased sweating in the finger; this trend was present until the end of the experimental trial. However, what is the most interesting, in the cited study phasic SCL activity in the forehead was observed during the experimental trial (compared to none during baseline measurement). This activity – and only this of all of the measured physiological responses – was proven to be associated with the experience of nausea.

The authors (Gavgani et al., 2016) give an interesting interpretation of their findings, which may shed new light on the physiological components of the simulator sickness experience. Some of the physiological symptoms (initial tachycardia, tachypnoea, finger sweating) were present at the initial phase of the VR exposure, in the time during which no self-reported nausea was present. This conclusion is supported by the fact that the above mentioned effects (except for finger sweating) became non-significant on the last, third exposure. The authors conclude that these symptoms may be evoked by emotions and arousal connected with the novelty of the VR experience. The forehead sweating, however, is related to the development of nausea. These results correspond with Treisman’s (1977) evolutionary theory of motion sickness – reducing the body temperature by increasing sweating serves as a survival strategy during intoxication.

##### Central nervous system

As a measure of the central nervous system activation, EEG has been used in some of the studies (Min et al., 2004; Chung et al., 2007). According to the results obtained by Min et al. (2004), there are significant differences in brainwaves patterns between rest and driving in a driving simulator. Such results have been obtained both for the frontal (Fz) and parietal lobe (Cz), giving similar patterns. After 5 min of simulator exposure, the δ/total increased and α/total, ß/total and 𝜃/total decreased significantly in 5–35 min of simulator exposure. Furthermore, the δ/total at Fz correlates positively, and both 𝜃/total and ß/total at Fz and Cz negatively, with the total SSQ score. The correlation with the SSQ score was the strongest for the 𝜃/total parameter (*r* = -0.842 at Fz and *r* = -0.93 at Cz), therefore the authors of the study (Min et al., 2004) propose that it could serve as the most effective physiological indicator of simulator sickness occurrence. This proposal was also supported by Chung et al. (2007).

#### Behavioral Measures – Postural Stability Tests

When relying on the Postural Instability Theory (Riccio and Stoffregen, 1991), one could use a postural stability test in order to assess the lack of postural stability as a specific manifestation of simulator sickness. Mourant and Thattacherry (2000) report using such test in their study. It is a simple and brief method – the person is asked to stand on the leg of their choice for 30 s in two separate trials. The time of standing without putting the other leg down is recorded and can be compared to the results of the same test after experimental manipulation or can serve as an independent measure. Although this method does not give a broad insight into simulator sickness symptoms, it can be useful when assessing changes in postural stability dependent on simulator exposure.

Cobb et al. (1999) report using a more complex set of postural stability tests: in their research program, the following methods of measurement were used: measuring the extent to which a static posture could be held, measuring the extent of hip sway over a 30 s period, walking on the floor and navigating over an uneven path with open eyes. Additionally, the authors administered two scales: task difficulty scale and subjective postural stability scale (Postural Stability Questionnaire – PSQ; Hamilton et al., 1989) after completing all the tasks.

### Temporal Aspects of Simulator Sickness

Questions regarding the temporal characteristics of the virtual reality experience which influence simulator sickness seem to recur in many papers (e.g., Kennedy et al., 2000; Moss and Muth, 2011; Domeyer et al., 2013). Although no unambiguous answers have yet been provided, some useful and promising leads can be found in literature and will be discussed herein. Since the main goal of the present work was to review research on simulator sickness from the temporal perspective, we decided to focus on research regarding one (or more) of the three issues described below.

As Kennedy et al. (2000) have observed, there are two main phenomena regarding the temporal aspect of simulator sickness: that the severity of simulator sickness increases with the increase of exposure duration during a single session, and that subjecting a person to several repeated simulator exposures may result in adaptation to the simulator conditions and thus in decrease of simulator sickness symptoms severity. The aforementioned aspects will be discussed in the present paper, as they seem to be crucial as far as virtual reality development is concerned. Furthermore, according to some research (e.g., Moss and Muth, 2011; Biernacki and Dziuda, 2014; Malińska et al., 2014), the simulator sickness symptoms appear to persist for some time after the simulator exposure – this aspect will be discussed below as well.

## Materials and Methods

### Search Strategy

A search of literature was performed in three electronic databases (Web of Science ‘all databases,’ PsychArticles, Scopus) with no publication date restriction. Since temporal aspects of simulator sickness rarely are the main focus of studies, we decided to retrieve a wide range of articles using the broadest term “simulator sickness” and assuming intensive article selection in subsequent stages. Thousand two hundred records were obtained. The search was conducted on 19th April 2018.

### Study Selection

Authors conducted a title and abstract screening, in order to exclude obviously irrelevant articles. Following keywords were used: *time, temporal, durat^∗^, adapt^∗^, persist^∗^*. The articles which titles and abstracts suggested an irrelevant area of research were excluded on this basis (1086 records). In the second stage of the screening process, full texts were retrieved and duplicated records removed (34 records). For 10 records full texts were unavailable and thus these records were excluded from the database as well. 70 articles were retrieved and evaluated in full text using the following criteria:

(1) published in full in English or Polish,
(2) based on empirical data,
(3) temporal aspects of simulator sickness are investigated,
(4) at least three time points for measurement of simulator sickness (applicable for studies regarding the temporal trajectory of the progression of simulator sickness),
(5) the study subjects were human,
(6) not investigating an intervention on simulator sickness,
(7) testing simulators or other forms of virtual reality (not 3D movies or desktop applications),
(8) measuring simulator sickness using psychometric methods (questionnaires).

After this process, 30 articles were retrieved. The authors decided to add 5 articles on the basis of hand search and previous knowledge. The final database consisted of a total of 35 articles (41 studies). A flow chart describing the search and screening process is presented in Supplementary Figure S1.

## Results

### The Temporal Trajectory of the Progression of Simulator Sickness

Studies on simulator sickness have been conducted since 1990s, using a wide array of virtual reality devices. Therefore, it is important to emphasize the fact that direct comparisons between studies using different hardware should be treated with extreme caution. Some trends may be observed, but it should be always borne in mind that for different devices and scenarios the temporal patterns of simulator sickness may vary significantly. Moreover, as some of the cited studies have been conducted almost 20 years ago, caution should be taken while making conclusions. However, the insight provided by the researchers appears to be valuable – while the technological development might have solved some of the problems, the methodology and qualitative conclusions are worth knowing.

In one of the studies conducted by Cobb et al. (1999), four subjects were immersed in a virtual reality environment for 1–2 h. Simulator sickness severity was measured with the Simulator Sickness Questionnaire. The participants were asked to remain in the virtual reality for up to 2 h. All participants reported the severity of symptoms increasing up to 1 h of exposure. Two of the participants withdrew after an hour when the simulator sickness symptoms experienced by them were too severe (mean scores for nausea: *M* = 67, oculomotor disturbance: *M* = 57 and disorientation: *M* = 82). The remaining two participants completed the 2-h immersion and reported that after 75 min the severity of symptoms decreased greatly. This suggests that although the simulator sickness symptoms severity increases with time, for some individuals it may be possible to adapt to the VR environment during a single exposure. Unfortunately, the sample in the study was too small to provide information on statistical significance of these effects. Nevertheless, these results are interesting and worth being taken into consideration when planning further experiments on extended VR exposure.

Kennedy et al. (2000) examined SSQ data from a military pilots’ flight simulator training database and categorized them by exposure duration into four categories (0–1, 1–2, 2–3 h, 3 or more hours). An analysis of variance revealed that the mean SSQ scores increase gradually when exposure duration increases. This trend proved to be statistically significant. No information on statistical significance of differences between each of the categories was given and it also should be noted that the analyzed data concerned many different simulator environments. It was also a between-subject design, therefore no conclusions about individual temporal patterns of simulator sickness severity can be made.

Min et al. (2004) have tested various measures of simulator sickness severity. In their study, both physiological and self-report methods were used – the Simulator Sickness Questionnaire was used for assessing the subjective severity of the syndrome. Only the results of the psychometric measurement will be reported herein. After baseline signal measurement and pre-experiment SSQ administration, the participants of the study drove a car simulator for 60 min, during which physiological measurements were conducted and the SSQ was completed orally after every 5 min of the simulator exposure, as well as after completing the whole trial. The authors of the study report that all of the participants showed symptoms of nausea, disorientation (after 10 min of simulator exposure) and oculomotor disturbance (after 25 min). The first significant difference between the baseline SSQ score and trial score appeared 10 min after beginning of the trial. The obtained results confirm the hypothesis that the severity of simulator sickness increases with time.

Moss and Muth (2011) tested several characteristics of HMDs as possible factors influencing simulator sickness severity, as well as the effect of a prolonged exposure. Only the latter of these effects will be reported herein. The participants’ task was to locate several objects in the virtual environment (a virtual laboratory), according to verbally given instructions, using only head movements. Each participant completed two practice sessions and five 2-min trials with 1-min breaks between them. A number of Simulator Sickness Questionnaire results were collected: before the experiment, after a practice session, after each trial, 5 and 10 min after the experiment. It was noted that the severity of simulator sickness symptoms increased with time – a significant effect of duration of the VR exposure was revealed. The most severe symptoms were noted after the last trial.

The type of walking interaction was the main topic explored by Lee et al. (2017), but their results also provide information about the temporal characteristics of simulator sickness. In their experimental design three types of walking control were included:

(1) a gamepad,
(2) sensors detecting hand movements and thus using specific hand gestures for walking control,
(3) a walk-in-place marching simulator with sensors and portable walking simulators attached to legs.

All of the participants of the study were exposed to three different VR environments (a cartoon town, a realistic nature environment and a low poly^2^ landscape in a three-step walking interaction: they either experienced them in the order of: gamepad, hand interface, walking simulator or in the reverse order – each of the participants completed nine VR experiences in total. The following variables were tested in the study: immersion, presence and simulator sickness (measured with the Simulator Sickness Questionnaire). The authors reported that the simulator sickness symptoms became more severe with time, although on the whole they were of moderate severity.

The above-mentioned study results support the hypothesis, that the severity of simulator sickness does increase with time during a single exposure, to various extents, which may differ depending on many variables (e.g., simulator type and its characteristics, length of the whole exposure, individual characteristics of the participants, etc.). Such results are confirmed in many other studies, which will be briefly summarized herein. Lo and So (2001) have confirmed that the nausea severity (measured by one question with answers ranging from 0 – “no symptom” to 6 – “moderate nausea, want to stop”) increases linearly with time during a 20-min exposure. Furthermore, the increase was significant in all of the comparisons, except for the one between the 15th and 20th minute of the trial. A similar study was conducted (So et al., 2001), and during a 30-min exposure the nausea ratings (measured in the same way as above) increased as well, but the differences were significant only in the 5th and 10th minute. Jarchow and Young (2007) have also measured the simulator sickness severity by asking just a single question (with a scale from 0 – “normal” to 20 – “about to vomit”). The subjects were tested on two consecutive days, as the main aim of the study was to assess the adaptation effect. It was however, discovered as well that within a single session the severity of symptoms increases, but this effect was observed in only one of the experimental conditions. In the study by Classen and Owens (2010), simulator sickness severity was measured at three time points: before VR exposure, after a 5-min acclimation exposure and after a 20-min trial. The obtained results indicated that the simulator sickness severity increased between the baseline score and both after-acclimation and post-exposure, but no significant differences were discovered between the after-acclimation and post-exposure scores. Therefore, one may presume, that the peak simulator sickness severity in this study was reached very early. However, no data was gathered during the 20-min exposure, so it is possible that some differences might have been discovered if more systematic simulator sickness measurements had been conducted. A similar procedure was conducted by Sinitski et al. (2018) – they measured the simulator sickness severity (with the SSQ) before the exposure, after an acclimation period (which lasted for 15 min) and after a 45-min trial. In this study, however, only a small increase in the disorientation scale was observed after the acclimation period and these symptoms decreased by the end of the session. Again, the period between the second and the third measurement was quite long, and therefore it is impossible to thoroughly analyze the pattern of the symptoms during the whole exposure.

An experiment conducted by Moss et al. (2008) consisted of a short practice and five 2-min experimental trials. It was confirmed that the simulator sickness (measured with the SSQ) severity increases with time – it was more severe after the last (5th) trial than: before the practice, after the practice, after the 1st, 2nd, and 3rd trials. As no significant differences were discovered between the 4th and 5th trial, it may be hypothesized that after circa 9 min of exposure the simulator sickness has reached its peak severity and would not become more unpleasant if the exposure duration was even longer. In a similar study (Moss et al., 2011), a phenomenon of the simulator sickness severity (measured with the SSQ) increase with the increased VR exposure duration was confirmed. Serge and Moss (2015) measured simulator sickness severity with the Revised Simulator Sickness Questionnaire and proved that it does increase with time when measured before VR exposure and after 8 and 16 min of exposure. Singer et al. (1998) report as well that the simulator sickness severity increases with time during a VR exposure, although the difference between a “Mid-Experiment” and “Post-Experiment” scores was not significant, suggesting an appearance of a threshold simulator sickness level. The authors, however, did not give information on how long the trials were, and therefore any conclusions drawn from this study should be treated with caution. Feenstra et al. (2011) have discovered a slightly different phenomenon than the ones above described – in their study, the differences in simulator sickness severity began to become statistically significant after the participants spent 10 min in the VR and then it increased until the end of the 20-min trial.

A systematic increase of simulator sickness severity (measured with the SSQ) with time was confirmed by Chung et al. (2007), Park et al. (2008), and Choi et al. (2009) during a 60-min trial and Aldaba et al. (2017), who measured simulator sickness severity with the SSQ, and by Reinhard et al. (2017), who used the Fast Motion Sickness Scale (FMS – a single-item scale, the scores on which range from 0 to 20). An increase of simulator sickness symptoms severity was also observed by McCauley et al. (1990), when it was rated on a 7-point scale (“normal, symptom-free” – “severe discomfort, I am unable to continue”) – it increased between measurement time points: before the exposure, in the middle of the 10-min task and after the whole 10-min task. There were 4 such trials and an increase in severity of the symptoms was observed for all of them. A brief summary of all reviewed studies is provided in the Supplementary Table S1.

Several conclusions can be drawn from the perspective of the temporal trajectory of the progression of simulator sickness on the basis of the studies retrieved. Firstly, there is empirical evidence to expect that severity of simulator sickness grows along time of exposure, as several studies using various approaches confirmed this hypothesis. In light of the reviewed research, this trend seems to be stable regardless of the technological progress in the field of VR presentation – the oldest studies (McCauley et al., 1990) and the most recent one (Sinitski et al., 2018) lead to the same conclusion. Even using between-subject comparisons leads – in most of the cases – to the conclusion that the severity of simulator sickness symptoms is greater when the exposure duration is longer (e.g., Kennedy et al., 2000). However, it is important to note that several moderators, which are not the main focus of this paper, may play a role here – for example, a simulator control method. Secondly, it is difficult to establish a universal rule regarding the maximum time individuals can spend in VR on the basis of the analyzed study results. On the other hand, in most of the studies the simulator sickness symptoms were experienced by all of the participants, not only the ones who reported some kind of tendency to feel sick.

Moreover, in some of the studies it was observed that the simulator sickness severity increases with time, but after reaching a certain level or after a certain amount of time it either begins to decrease (Cobb et al., 1999; Sinitski et al., 2018) or remains on the same level (Singer et al., 1998; Lo and So, 2001; So et al., 2001; Moss et al., 2008; Classen and Owens, 2010). It can lead to a conclusion, that during a single VR exposure it is possible for some people to achieve simulator sickness adaptation (or, for some simulator types, to evoke the adaptation effect). However, it should be further explored whether this effect transfers to subsequent VR sessions.

On the other hand, it has also been proved that in some cases the simulator sickness symptoms begin to show after some time spent in VR and that this time threshold may be different for various simulator sickness symptoms (Min et al., 2004; Feenstra et al., 2011). Although this type of evidence is less prevalent than the one described above, it is also worth being taken into consideration. If the symptoms start being unpleasant after some time, a single VR session should be short enough to prevent these symptoms from occurring.

Keeping in mind several moderators which may vary between software (e.g., way of control, setting, graphics quality), another strategy of testing temporal tolerance may be reasonable, viz. testing of certain VR software using precisely selected methods. In order to make it possible, various methods need to be integrated, and standardized methodology needs to be developed.

### Possibility of Adapting VR Users in Advance

As Nader and Kruszewski (2013) suggest, simulator sickness can be avoided when the virtual reality users are allowed a sufficient amount of time to adapt to the simulator conditions. They propose that such adaptation sessions may last for a number of days and involve an increase in time spent in the simulator during a single training, as well as an increased difficulty of the task. This proposal appears to be congruent with the assumptions of some of the theories. For example, according to the Neural Mismatch Model (Reason, 1978), unpleasant symptoms occur when the present sensory information is inconsistent with past experiences of the individual. Gaining such experience in the specific virtual reality environment might prevent the aforementioned conflict. Similarly, when one is allowed to immerse in virtual reality several times, one can learn how to maintain balance in such an environment – adaptation appears to be possible in the paradigm of the Postural Instability Theory (Riccio and Stoffregen, 1991) as well. It should also be emphasized that adaptation to simulator sickness in VR may be achieved not only by exposure to an identical virtual environment, but also by similar experiences, such as video gaming. It has been shown that individuals with more gaming experience and more self-reported “computer skills” experienced less unpleasant symptoms during a VR session (Häkkinen et al., 2006a). However, there are also studies which do not support this claim (e.g., Häkkinen et al., 2002, 2006b), therefore this issue needs further testing.

Some adaptation effects were observed by Lampton et al. (2000). In their study, five separate VR immersions were conducted (trainings 1 and 2 and missions 1, 2, and 3). The SSQ was administered before and after each immersion. The pre-post immersion score difference was significant for the first training and the second and third mission, and not significant for the second training and first mission. Therefore, it can be concluded, that after the first training the participants achieved some adaptation, but its effect wore off with time. Similarly, in the study by Domeyer et al. (2013), the adaptation effect was obtained during a series of VR exposures conducted on 1 day, and in this study the subjects did adapt to the simulator conditions (the effect was visible on the total Revised Simulator Sickness Questionnaire score). Such effects may occur even during a relatively short exposure, lasting 45 min in total (Sinitski et al., 2018). In the quoted study the participants experienced an increase in disorientation symptoms (measured with the SSQ) at first, but it decreased by the end of VR exposure. However, such effect was not confirmed for the remaining SSQ subscales and for the total score. Additionally, it should be stressed that all of the VR immersions of the two studies mentioned above took place during a single day, which is quite unusual for studies exploring adaptation effects – usually each of the immersions is conducted on a separate day.

In the study program developed by Cobb et al. (1999), 12 individuals participated in three consecutive virtual reality sessions, each of which lasted 20 min, with a 1-week break between the sessions. The simulator sickness symptoms severity (measured with the Simulator Sickness Questionnaire) decreased after each consecutive VR exposure, especially strongly for the disorientation symptoms, which is consistent with the results obtained by Sinitski et al. (2018). A similar effect of adaptation was observed by Braithwaite and Braithwaite (1990) and Bailenson and Yee (2006) – in their studies, the simulator sickness symptoms (measured with the SSQ) decreased in severity with time.

An interesting form of adaptation training was proposed by Smither et al. (2008). They tested the ability of a self-propelled rotation stimulation (SRS)^3^ to provide adaptation to simulator sickness. Ten subjects took part in five SRS trials on separate days and on the last day were exposed to a VR, and 10 other subjects took part only in the latter part of the experiment, providing a control group. The control group experienced significantly more severe dizziness symptoms and higher total, disorientation and oculomotor disturbance SSQ scores. These results show that adaptation can be achieved without immersing in the virtual reality, but some form of pre-immersion training is needed to prevent the unpleasant symptoms, as the participants from the control group, who did not have a chance to adapt in any form, suffered from the simulator sickness.

Kennedy et al. (2000) analyzed data collected from 53 individuals – military pilots, who participated in seven consecutive helicopter simulator trainings. A repeated-measures analysis of variance indicated that a monotonic decrease in simulator sickness severity (measured with the SSQ) as a function of flight number can be observed. Furthermore, for some subjects a floor effect was observed – they reached a total adaptation and the SSQ 0 score at some point, which did not increase in further trials. This effect is responsible for the deceleration in the decline of simulator sickness severity with time. The authors propose that, according to their results, short, repeated simulator exposures may be used in order to achieve adaptation to the VR environment and to prevent simulator sickness. Moreover, they further conclude that the decrease in simulator sickness severity after several trials exceeds the increase in severity with a single longer exposure duration.

Brooks et al. (2010) conducted two studies – an exploratory and a confirmatory one. In the exploratory study (a combination of results of three independent studies), the participants were immersed in a driving simulator. After a training session, four 5-min trials using slightly different conditions (e.g., a curvy road instead of a straight one) were conducted. Between the sessions, 2-min rest periods took place. Before and after each trial, the participants completed the Motion Sickness Assessment Questionnaire, the score of which served as an indicator of simulator sickness severity. In the confirmatory study the main difference was that the participants completed three 30-min experimental trials in the same simulator. The authors report that for some participants an adaptation effect was showed – their symptoms’ severity increased at first, but then decreased as they became accustomed to the simulator experience. No statistical parameters were provided to describe this tendency, but it still appears to be a promising information.

In a study by Newman et al. (2013) the subjects took part in 6 VR immersions, five of which happened on consecutive days and the last – 22 days after the first immersion. It was discovered, that the simulator sickness symptoms assessed on a 0–10 scale decreased rapidly after the first exposure – the comparisons were significant for Day 1 and each of the other times and not significant for any other comparisons. It appears that the adaptation achieved by the study subjects happened between the two first sessions. What is more, that adaptation effect did not wear off with time – on Day 22 the symptoms severity was still significantly smaller that on Day 1. The SSQ was also administered in this study and the total score, nausea and disorientation scores did significantly decrease in time. This effect, however, was visible between Day 1 – Day 4 and Day 1 – Day 5 (for the total and nausea scores) and between Day 1 – Day 4 (for the disorientation score). Furthermore, for the total and nausea scores, adaptation was retained during the last measurement on Day 22. The results of this study prove that it is possible to adapt people to VR conditions and that this effect can be long-lasting. However, the method of measurement for simulator sickness severity should be chosen cautiously, as the effects may slightly differ when using different methods. Probably the best option would be to use at least two reliable methods of comparison as it was done by Newman et al. (2013).

Helland et al. (2016) conducted an experiment on a driving simulator, during which the effects of simulator sickness, blood alcohol concentration and repeated simulator exposures on driving performance were studied. Herein, only the results concerning the relationship between repeated simulator exposures and simulator sickness severity will be discussed. A driving simulator consisting of the body of a car and three screens were used. The study included three 60-min long driving tests in the simulator (with at least 2-day breaks between the trials). After every trial each of the 20 participants assessed the simulator sickness severity by rating it on a scale from 0 to 10 – they were asked – “To what extent did you experience simulator sickness during the driving test?”. It is worth noting that the mean simulator sickness score was very low in this study (*M* = 2.5), which might have had an impact on the results. For the participants, who did not interrupt any of the sessions (*N* = 13), the mean simulator sickness severity score was 3.4 for the first, 1.8 for the second and 1.5 for the third session. Although the simulator sickness severity appears to decrease with consecutive sessions, the relationship was not statistically significant. It could be hypothesized that had the authors used a more precise method for assessing the simulator sickness severity, the results could have been different. With the concise, one-question simulator sickness severity measurement, the data given in the study report do not fully support the hypothesis that simulator users adapt to the virtual reality conditions.

Another study providing evidence supporting the hypothesis, that simulator sickness adaptation is possible, was conducted by Reinhard et al. (2017). Twenty eight participants took part in the experiment, it had two parts, separated by 7–14 days of a break. On the first day, six 20-min drives in a simulator took place and on the second day there were four of them. To assess the simulator sickness severity, two scales were used: the FMS and the SSQ. The authors report an interesting pattern of results. During both sessions, the severity of symptoms did increase, but that increase was less visible during the second session. Thus, an adaptation effect was proved, but it was not a complete disappearance of symptoms. It was stressed in the paper, that the first VR immersion should be treated with extreme caution – the subjects should be monitored for unpleasant symptoms, the rests between trials should be longer and the trials themselves shorter than usual. For a summary of studies reviewed in this aspect, see the Supplementary Table S2.

In light of the reviewed studies, the possibility of adapting to VR is reasonable – several authors reported results suggesting it. However, a large number of the studies did not report statistical tests proving this claim or reported statistical non-significance. Various adaptation patterns have been observed – the effect was visible when all of the VR immersions were conducted on a single day (Lampton et al., 2000; Domeyer et al., 2013), on separate days (e.g., Cobb et al., 1999; Brooks et al., 2010; Reinhard et al., 2017), or even during a single VR exposure (Sinitski et al., 2018). A floor effect of no symptoms after some exposures was observed by Kennedy et al. (2000). The effect of adaptation does not wear with time, as in was observed by Newman et al. (2013). Furthermore, virtual reality is not necessarily essential for evoking the adaptation effect (Smither et al., 2008).

The patterns and extents to which adaptation was observed in the aforementioned studies are diversified. Certainly, further research on this issue is necessary. It is also intriguing what is the relationship between possible adaptation along with subsequent VR experiences and increasing severity of simulator sickness during one long experience. These relationships would be worth testing in future studies.

### Persistence of the Simulator Sickness Symptoms After VR Exposure

Tanaka and Takagi (2004) discovered, that not only the simulator sickness symptoms persist for some time after VR exposure, but also the length of the persistence is dependent on the initial symptom severity. For the participants who suffered from severe symptoms (total SSQ score of more than 60), the recovery time was longer than 30 min. On the other hand, the subjects, who experienced only slight symptoms (total SSQ score of 25 or less) needed no longer that 5 min to recover from the simulator sickness symptoms.

In the study by Bos et al. (2005) it was also confirmed that the simulator sickness symptoms tend to persist for some time after VR exposure, but they return to baseline [a score of 0 on the Misery Scale (MISC); the maximum score on this scale is 10] in an hour following the end of the VR exposure for most of the participants. Only 4 of 24 subjects did not fully recover within 2 h post exposure, with the maximum MISC score of 3. These conclusions are supported by the results obtained by Keshavarz et al. (2018). In their study simulator sickness was measured using the FMS and 36 of 121 participants were forced to drop out before the end of the experimental task. The total time until recovery (operationalized by a FMS score of 1 or less) between the participants who finished the task and those who dropped out earlier varied significantly – the latter needed more time to recover. However, only five subjects (all from the drop-out group) did not fully recover 15 min post exposure. Furthermore, for all of the participants there was a significant decrease of simulator sickness symptoms severity between immediately after exposure and 3 min later. Results achieved by Singer et al. (1998) support the hypothesis that the simulator sickness symptoms persist for some time after leaving the VR and then return to the baseline levels. In their study, all of the specific symptoms except disorientation (viz. nausea and oculomotor disturbance; the same effect was confirmed for the total SSQ score as well) returned to baseline levels after a 30-min rest. McCauley et al. (1990) state that the simulator sickness symptoms severity decreases after leaving the VR (between two measurement points: immediately after leaving the VR and 30 min later).

A more detailed, qualitative description of the simulator sickness symptoms persistence pattern was given by Braithwaite and Braithwaite (1990). From 14 of the participants, 6 suffered from severe headaches, which lasted for 2-6 h, 2 suffered from nausea (up to 2 h after leaving the simulator) and 6 participants reported experiencing other symptoms, which cannot be classified as typical simulator sickness symptoms (visual flashbacks, unsteadiness or symptoms different from the ones experienced during the VR exposure). Unfortunately, no information on the VR exposure length was given by the authors.

In the study by Moss and Muth (2011), more widely described above, it was discovered that the simulator sickness symptoms persist for some time after leaving the virtual reality environment. The total SSQ score in this study measured 10 min post exposure was still significantly higher than the baseline score. This means, that for the virtual reality environment tested in the study, not only did the simulator sickness’ symptoms increase with time, but they also persisted for at least 10 min after leaving the virtual reality. Therefore, it cannot be confirmed when did the symptoms subside. However, in a similar study by Moss et al. (2011), the severity of symptoms did return to baseline level after a 10-min rest.

Biernacki and Dziuda (2014) have studied simulator sickness symptoms on a group of professional truck drivers, who participated in three 30-min truck simulator drives – the first one on a fixed-base platform with poor visibility (created by a simulated fog) and twice with good visibility: on a fixed base and on a mobile platform. The simulator consisted of a truck cabin and a cylinder screen, on which all visual stimuli were displayed. The simulator sickness was measured with the Simulator Sickness Questionnaire. The questionnaire was completed five times for each exposure: before each trial, 2 and 30 min after all of the trials, in the evening of the same day and next day, in the morning. The level of nausea, disorientation and oculomotor disturbance, as well as the total severity of simulator sickness symptoms proved to be dependent on the measurement time point. The level of nausea was higher 2 min than 30 min after exposure. The time profile for oculomotor disturbance, disorientation and the total SSQ score turned out to be similar: the scores 2 min after exposure were significantly higher than 30 min after exposure and the baseline scores. The symptoms of simulator sickness seem to retreat after leaving the virtual reality environment, but only for the nausea factor the simulator sickness severity 30 min post exposure did not differ significantly from the baseline score. Half an hour appears not to be sufficient time for the symptoms to disappear completely. In another paper (Dziuda et al., 2014) describing the results of this study, the authors state, that the severity of nausea measured 2 and 30 min post exposure and in the evening of the same day was significantly higher than in the morning of the next day.

Malińska et al. (2014) tested subjective sensations (simulator sickness and fatigue; the latter will not be discussed herein) felt after exposure to virtual reality. In this study, individual proneness to motion sickness was tested using the Coriolis test before the experimental trial. Twenty men participated in the experiment. The study was conducted in two separate phases. During the first phase, all of the participants watched a part of the “Avatar” movie – both in 2D and 3D versions. The results concerning only the impact of the movie will not be discussed herein. In the second phase, the participants engaged in a virtual reality task, which included transporting various elements on a virtual workstation. A questionnaire created by the authors of the study was used as a method of measurement for the simulator sickness. It included 8 symptoms (e.g., eye pain, headache, dizziness, nausea), which were assessed on a five-point scale. This questionnaire was conducted thrice – straight after the simulator exposure, 20 min and up to 24 h later (and sent by email). 20 min post exposure, 7 of 8 simulator sickness symptoms were reported by at least one participant. No one experienced increased sweating and the most prevalent symptoms were: eye pain, drowsiness, fatigue and apathy. According to the results, the participants experienced the simulator sickness symptoms up to 4 h after completing the virtual reality task. Reported symptoms included: headache, dizziness, disorientation and drowsiness. Unfortunately, no comparison between the different time periods was given, and therefore any conclusions drawn from this study regarding the temporal aspects of simulator sickness should be treated with extreme caution. The results of the studies concentrated on simulator sickness persistence are given in the Supplementary Table S3.

Regarding simulator sickness persistence, it may be assumed that at least some of the symptoms may prevail after the exposure (10 min, Moss et al., 2011; circa 30 min, Singer et al., 1998; more than 30 min, Biernacki and Dziuda, 2014; Dziuda et al., 2014), in some cases even for relatively long time (more than 4 h after approximately 2 h of exposure, Malińska et al., 2014; for even 4 h after leaving the VR, Braithwaite and Braithwaite, 1990). On the other hand, the results of Biernacki and Dziuda (2014) suggest that the severity of symptoms changes rapidly – it is increased directly after exposure, but significantly decreased 30 min afterward. The time of the symptoms’ prevalence differs between various VR environments. Furthermore, the length of recovery depends on the initial symptoms’ severity – it takes longer to fully recover, when the experienced symptoms were more severe (Bos et al., 2005; Keshavarz et al., 2018).

## Conclusion

To summarize the conclusions reached about each of the temporal aspects of simulator sickness, a sufficient amount of evidence appears to exist in order to confirm the hypothesis that the severity of simulator sickness symptoms increases with increased exposure time. There appears to be no universal rule regarding maximum exposure time until unpleasant symptoms are evoked. A correct direction of research in this aspect would be to test the temporal pattern of simulator sickness progression for each VR technology separately – as it has been reported by Lee et al. (2017), different devices used for controlling the individual’s movement in the virtual environment tend to evoke slightly different levels of simulator sickness. Despite the development of technology, the issue of simulator sickness appears to still remain unsolved. Interesting trends have been reported – in some studies, the simulator sickness severity either begins to stabilize (e.g., Moss et al., 2008) or decreases (e.g., Sinitski et al., 2018) after some time and in other – the symptoms become noticeably unpleasant after some time spent in the VR (e.g., Min et al., 2004). As it has been broadly discussed above, adaptation to the VR environment appears to be possible, but the quoted studies do not provide conclusive data – further inquiry regarding this topic is necessary. Some simulator sickness symptoms may prevail for some time after exposure, although it remains unknown for how long and it may vary depending on the initial severity of the symptoms.

Apart from the points concerning each specific temporal aspect of simulator sickness, some general conclusions can be drawn. The virtual reality technology and simulators still have the tendency to evoke unpleasant symptoms among their users; although the technology advances, this problem has not yet been solved. It is the most vivid for the first aspect discussed herein – the temporal trajectory of the progression of simulator sickness – the severity of symptoms grows along exposure time both in the studies conducted almost 20 years ago (Cobb et al., 1999) and in the most recent ones (Lee et al., 2017). Although this trend appears to be stable regardless the technological progress, such statements should be treated with caution, as the studies used various types of VR technologies, which may not be comparable.

Until the technology reaches the point when the simulator sickness will be wholly preventable, some standards should be developed when it comes to research on virtual reality and simulators. The issue of how often the simulator sickness symptoms should be measured (not only during the experimental trial, but also after it), should be addressed.

It would be advisable to test the tendency of a new virtual reality tool to evoke the simulator sickness symptoms in the three above discussed dimensions: temporal pattern of the symptoms’ progression, adaptation possibility and persistence of symptoms after exposure. These parameters would provide vital information on how long the training, game or any other scenarios should be, in order to provide the user with an enjoyable experience and to prevent unpleasant sensations. This issue appears to be exceptionally crucial for professional training simulators, where the quality of the experience may have an influence on results of the training session. Furthermore, the physiological measurement of simulator sickness should be developed and given more focus, as it might be more precise and less biased than a self-report.

The researchers and developers employing the virtual reality technology should always bear in mind the fact that simulator sickness exists and can disturb the desired outcomes. Therefore, before it becomes widely implemented, every VR technology needs to be tested for its tendency to evoke unpleasant symptoms in its users in the three temporal aspects discussed above.

### Practical Implications for Further Research

The above described research provides interesting insight into the temporal aspects of the simulator sickness and it appears that there are still issues which demand further inquiry. First of all, most of the research concerns driving or flight simulators, most often used for training professional drivers and pilots, but the virtual reality technology is advancing rapidly and has already been applied to the gaming industry (2.704 titles on Steam^4^ when the searching parameters were restricted to “VR only” and 3.243 with the “VR supported” search restriction; data collected on June 14, 2018) – creating a brand-new field for research. It would be advisable to explore the temporal aspects of simulator sickness, not only on professional training simulators and professional drivers and pilots, but also on virtual reality-supported games and everyday, non-professional VR users and gamers.

It would also be advisable to further explore the temporal aspects of simulator sickness and to develop a standardized methodology which would allow a comparison between studies focusing on different virtual reality environments. Researchers should bear in mind the need to compare the SSQ scores between time periods [a good example of such methodology is the Moss and Muth (2011) study, where simulator sickness severity was assessed each 5 min] and to control the severity of symptoms for several hours after virtual reality exposure, in order to be able to determine the moment when the symptoms subside.

Moreover, it would be intriguing to compare the effect of one prolonged VR exposure to a number of shorter exposures, summing up to the same total time. According to the evidence found in past studies, it could be expected that the severity of symptoms after one long exposure should be greater than after a series of short ones. A pattern of symptoms’ persistence after such two types of exposures could also be explored.

It is also worth suggesting that the simulator sickness severity should be assessed not only before the experimental procedure, but also after the initial training phase, in order to establish if the training could serve as the adaptation period.

In light of the past research which suggest that most of the people suffer from simulator sickness to some extent, the researchers should care for the study participants, who report strong and unpleasant symptoms not only straight after the experimental procedure, but also as long as the symptoms persist. Brooks et al. (2010) propose a number of means that can be taken in order to provide the participants with proper care. Supplies such as sick bags, plastic gloves, mouthwash and cleaning products should be kept in the lab. The participants should be provided with light snacks and water. They should also be advised not to drive a car until they feel that all the symptoms have subsided. Brooks et al. (2010) suggest as well that the participants should stay in the lab for at least an hour after the experiment. It would also be advisable to contact the participants after the study and ask them if they experienced any unpleasant side-effects of VR exposure.

### Strengths and Limitations

The main strength of the present paper is that it covers a very wide array of study reports – not only from the most recent times, but also the older ones, from the 1990s. Consideration has been taken to analyze all the results thoroughly. Caution has been exercised to allow for any possible bias and limitations of every single study. Moreover, efforts have been taken to shed more light on the subject which, despite being an important factor of simulator and VR experience, has not been given much attention in research.

A significant number of the reviewed studies turned out to have drawbacks or did not include as thorough analysis of the temporal aspects of simulator sickness as it may have been expected, which can be considered a limitation of the present review. Very often the study reports did not include any information on statistical significance of the results, or the sample size was extremely small, which made it impossible to draw definite conclusions. Furthermore, as the temporal aspects of simulator sickness is most often analyzed alongside other study objectives, it is possible that some interesting results on the topic have been omitted in the search process. Despite these limitations, the present review is believed to give insight into the temporal aspects of simulator sickness and serve as a basis for further research focused on temporal aspects of simulator sickness.

## Author Contributions

ND wrote major part of the paper, contributed to the conception and design of the review. PS designed the review and wrote minor part of the paper. AS contributed to the conception and design of the review. All authors listed have made substantial intellectual contribution to the work, revised the manuscript, read and approved the submitted version.

## Conflict of Interest Statement

The authors declare that the research was conducted in the absence of any commercial or financial relationships that could be construed as a potential conflict of interest.

## Footnotes

1 Optokinetic nystagmus – an eye pursues a target object from one end of a visual field to the other. When the eye can pursue the object no further, it snaps back to the far side of the visual field where it begins to pursue again. Vestibular ocular response – responsible for keeping a target object on the fovea, the center of the retina where one’s vision is sharpest, when the head is turning.

2 Consisting of a small number of polygons.

3 “In the SRS, participants were asked to raise their right hands above their heads and grasp their right earlobe with their left hand, bend at the waist, and spin in a clockwise direction under self-propelled condition. The participants spun 10 times in 30 s (20 RPMs) and this constituted a trial” (Smither et al., 2008, pp. 330–331).

4 Steam (https://store.steampowered.com) is a digital distribution platform, on which various types of games can be bought, played and stored in a cloud.

## Supplementary Material

FIGURE S1 Flow chart of the search and screening process for the relevant literature.

TABLE S1 Studies focusing on the temporal trajectory of the progression of simulator sickness.

TABLE S2 Studies focusing on the possibility of adapting VR users in advance.

TABLE S3 Studies focusing on how long the simulator sickness persists after VR exposure.
