# Supplementary figures and images for: Can Simulator Sickness Be Avoided? A Review on Temporal Aspects of Simulator Sickness
Source: Front Psychol. 2018 Nov 6;9:2132. doi: 10.3389/fpsyg.2018.02132 (PMC6232264; doi:10.3389/fpsyg.2018.02132)

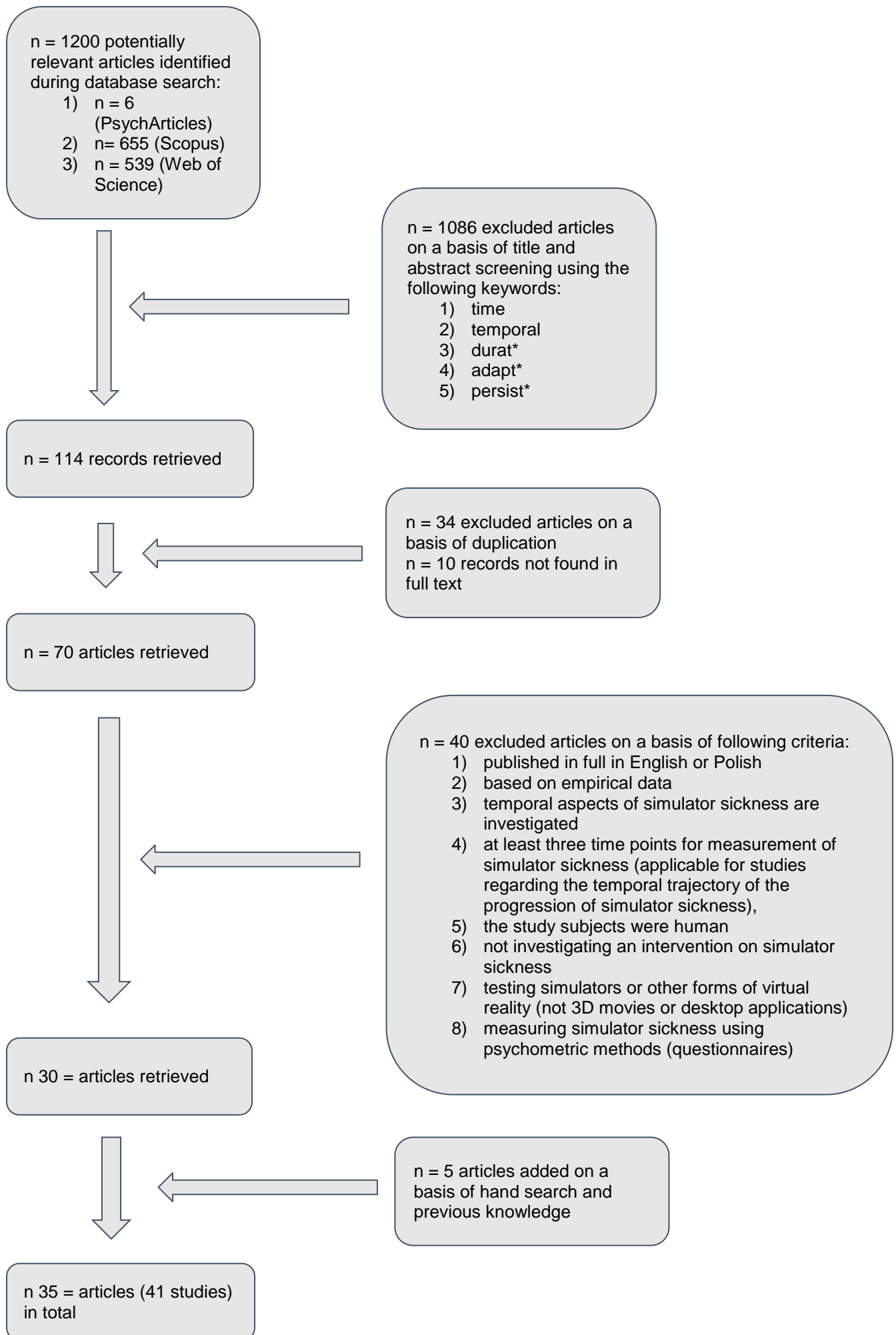

Supplement: FIGURE S1 — Flow chart of the search and screening process for the relevant literature. [file Data_Sheet_1.pdf]
