# Supplementary material for: Can Simulator Sickness Be Avoided? A Review on Temporal Aspects of Simulator Sickness
Source: Front Psychol. 2018 Nov 6;9:2132. doi: 10.3389/fpsyg.2018.02132 (PMC6232264; doi:10.3389/fpsyg.2018.02132)
Supplement: TABLE S1 — Studies focusing on the temporal trajectory of the progression of simulator sickness. [file Data_Sheet_2.PDF]

The temporal trajectory of the progression of simulator sickness

Authors and publication date	Study design (between or within subject)	Number of participants	Type of virtual reality technology used	Time of simulator/VR exposure	Method of measurement	Periods when simulator sickness was measured	Results (concerning the temporal aspect)
Cobb et al., 1999	between subject	4	Provision system, a virtual gallery	1 or 2 hours	Simulator Sickness Questionnaire	Non-specified	All participants: SSQ scores increasing up to one hour. Two participants: withdrawal after one hour, severe simulator sickness symptoms. Two participants: after 75 min symptoms subsided.
Kennedy, Stanney, Dunlap, 2000	between subject	938	Military flight simulators	4 categories: 0-1 hour, 1-2 hours, 2-3 hours, 3 or more hours	Simulator Sickness Questionnaire	Non-specified	SSQ scores increase gradually when exposure duration increases.
Min et al., 2004	within subject	20	Driving simulator	60 minutes	Simulator Sickness Questionnaire, EEG, GSR, ECG, skin temperature	SSQ: before the experiment, 12 times during the experiment (every 5 minutes; orally), after the experiment. = 14 times in total	Total SSQ score and scores for all subscales increase significantly in time. Total SSQ, disorientation, nausea - significant increase from the 10th minute of trial. Oculomotor disturbance - significant increase from the 15th minute of trial.
Moss, Muth, 2011	between subject	80	HMD; locating objects in VR (head movements)	five 2-min trials	Simulator Sickness Questionnaire (orally; pre-recorded cassette)	SSQ: before the experiment (pre-practice), after the practice sessions, after each trial (5 times), 5 min post exposure, 10 min post exposure = 9 times in total	A significant effect of VR exposure duration. After 5th trial - significantly higher SSQ total score that all other measurements.
Lee, Kim, Kim, 2017	within subject	20	Three types of walking simulators: HMD + a gamepad (Xbox 360); Only HMD + sensors detecting hand movements; HMD + march-in-place detectors: sensors and portable	Not given in detail; three trials in each environment = 9 trials in total	Simulator Sickness Questionnaire	After each trial = 9 times in total	Simulator sickness symptoms severity increases in time (between 180 s and 300 s exposure duration) - the change was the biggest for the gamepad, the smallest for the march-in-place device (but no information on statistical significance).

			simulators attached to legs. Three VR environments (cartoon town, realistic nature environment, low poly landscape).				
Lo and So, 2001	within subject	16	HMD	20 min, 4 separate days (at least 7 days break)	Nausea severity on a 0-6 scale (“no symptom” – “moderate nausea, want to stop”) Simulator Sickness Questionnaire	Nausea – verbally, each 5 min SSQ – before and after exposure	Nausea severity increases linearly during a 20-min exposure (no significant difference between the 15th and 20th minute).
So, Lo, Ho, 2001	within subject	96	HMD (Virtual Research VR4)	30 min	Nausea severity on a 0-6 scale (no symptom – moderate nausea, want to stop) Simulator Sickness Questionnaire	Nausea – verbally, each 5 min SSQ – before and after exposure	Nausea scores increase significantly between the 5th and 10th minute of the exposure.
Jarchow and Young, 2007	within subject	Not given	HMD	Two consecutive days, each time circa 12 min	Single question, 0-20 scale (“normal” – “about to vomit”)	Once per minute	Within a single session the severity of symptoms increases (only in one of the conditions).
Classen and Owens, 2010	within subject	44	Not given	Circa 25 min	Simulator Sickness Questionnaire	Before the exposure, after an acclimation period, after a 20-min trial.	The simulator sickness severity increases between the baseline score and after-acclimation and between baseline and post exposure (not between after-acclimation and post exposure).
Sinitski et al., 2018	within subject	30	CAREN-Extended VR (a curved visual projection display + a treadmill)	60 min	Simulator Sickness Questionnaire	Before the exposure, after a 15-min acclimation period, after a 45-min trial.	A small increase in the disorientation scale observed after the acclimation period; symptoms decreased by the end of the session.
Moss, Scisco and Muth, 2008	within subject	10	HMD	Two sessions separated by 7 days: two 48-s practice trials + five 2-min trials = circa 12 min during	Simulator Sickness Questionnaire	Before the practice, after practice, after each of the 5 trials	Simulator sickness severity increases in time (more severe after the last trial than: before the practice, after the practice, after the 1st, 2nd and 3rd trials). No significant differences discovered

				single session			between the 4th and 5th trial.
Moss et al., 2011	within subject	22	HMD	Two sessions separated by 7 days: two 48-s practice trials + five 2-min trials = circa 12 min during single session	Simulator Sickness Questionnaire	Before the practice, after practice trials, after each trial, 5 and 10 min post exposure	Simulator sickness severity increases with increase of exposure duration.
Serge and Moss, 2015	within subject	12	HMD – Oculus Rift	16 minutes	Revised Simulator Sickness Questionnaire	Before VR exposure, after 8 and 16 minutes of exposure	Simulator sickness severity increases in time.
Singer, Ehrlich and Allen, 1998	within subject	32	Virtual Research Corporation VR4 HMD	Not given	Simulator Sickness Questionnaire	Before VR exposure, “Mid-Experiment”, “Post-Experiment”, after 30 min rest	Simulator sickness severity increases in time, difference not significant or the “Mid-Experiment” and “Post-Experiment” comparison.
Feenstra, Bos and van Gent, 2011	within subject	11	Motion simulator – a moving cabin with screens	3 days in a row, each day 20 min	Misery Scale (MISC)	Before the immersion, and at five time points: 2nd, 5th, 10th, 15th and 20th minute.	Simulator sickness severity increases in time, but after spending 10 minutes in the VR.
Chung et al., 2007<br>Park et al., 2008<br>Choi et al., 2009	within subject	20	Driving graphic simulator	10 min practice + 60 min trial	Simulator Sickness Questionnaire	Every 5 min	Simulator sickness severity increases in time.
Aldaba et al., 2017	within subject	20	HMD: HTC Vive or Oculus Rift CV1	4 sessions with at least 1 day break single trial time not specified	Simulator Sickness Questionnaire	Before the VR immersion and after the 1st, 4th and 8th trial	Simulator sickness severity increases in time.
Reinhard et al., 2017	within subject	28	Fixed-base driving simulator with a projection screen and a cockpit of a car	Two days, 7-14 days of break First day: 6 20-min immersions. Second days: 4 20-min immersions	Fast Motion Sickness Scale	Every minute	Simulator sickness severity increases in time.
McCauley et al., 1990	within subject	48	Flight simulator – motion system, a cockpit, screen	10 min training + 4 10-min trials	A 7-point scale (“normal, symptom-free” – “severe discomfort, I am unable to continue”)	Three times during each trial: before, in the middle, after	The severity of symptoms increases between the measurements.
