# Supplementary material for: Can Simulator Sickness Be Avoided? A Review on Temporal Aspects of Simulator Sickness
Source: Front Psychol. 2018 Nov 6;9:2132. doi: 10.3389/fpsyg.2018.02132 (PMC6232264; doi:10.3389/fpsyg.2018.02132)
Supplement: TABLE S2 — Studies focusing on the possibility of adapting VR users in advance. [file Data_Sheet_3.PDF]

Possibility of adapting VR users in advance

Authors and publication date	Study design (between or within subject)	Number of participants	Type of virtual reality technology used	Time of simulator/VR exposure	Method of measurement	Periods when simulator sickness was measured	Results (concerning the temporal aspect)
Lampton, Rodrigues, Cotton, 2000	within subject	93	HMD	Not given in detail, 5 immersions during one day: training 1, training 2, mission 1, mission 2, mission 3	Simulator Sickness Questionnaire	Before and after each immersion: training 1, training 2, mission 1, mission 2, mission 3.	The pre-post immersion score difference significant for training 1 and mission 2, training 1 and mission 3; significant for the training 2 and mission 1. Some adaptation achieved after the first training (effects wearing off in time).
Domeyer, Cassavaugh and Backs, 2013	within subject	120	Driving simulator: a screen, motion base, body of the car	10 min acclimation period + 11 scenarios (time not given in detail) – all during one day	Revised Simulator Sickness Questionnaire	Before the acclimation and after the tasks	Adaptation is possible during a series of VR exposures on one day.
Sinitski et al., 2018	within subject	30	CAREN-Extended VR (a curved visual projection display and a treadmill)	60 min	Simulator Sickness Questionnaire	Before the exposure, after a 15-min acclimation period, after a 45-min trial	Increase in disorientation scale after acclimation period; symptoms decrease by the end of the session.
Cobb et al., 1999	within subject	12	Elysium system, passive VR environment (hospital corridors)	Three 20-min sessions	Simulator Sickness Questionnaire	Non-specified (after each exposure?)	SSQ scores decreased significantly between sessions 1 and 2, 2 and 3 (especially strongly for disorientation symptoms).

Bailenson and Yee, 2006		within subject	9	HMD – Virtual Reseach VR or nVisor SX HMD	3 sessions, each lasting 35-40 min, spread across 10 weeks	Simulator Sickness Questionnaire	After each session	Adaptation is possible during a series of VR exposures.
Braithwaite and Braithwaite, 1990		between subject	115	Helicopter simulator – with the body of a helicopter and screens	Not specified in detail, different across participants	Simulator Sickness Questionnaire	After a session	Adaptation is possible during a series of VR exposures.
Smither, Mouloua and Kennedy, 2008		between subject	10	HMD – Virtual Research V6	First group – 5 trials of self-propelled rotation stimulation on separate days, on the last day – VR (20 min). Second group – only the VR part (20 min)	Simulator Sickness Questionnaire	After the SRS trials; after VR trial – 0, 15, 30, 45 and 60 min after VR immersion	Adaptation to the VR with the self-propelled rotation stimulation is possible. Control group – more dizziness symptoms; higher total, disorientation and oculomotor disturbance SSQ scores.
Kennedy, Stanney, Dunlap, 2000		within subject	53	Military helicopter simulator	7 sessions; the time of a single session non-specified	Simulator Sickness Questionnaire	After each session	Monotonic decrease in SSQ scores as a function of session number. Floor effect (caused by 0 scores in SSQ) – deceleration in the decline of SSQ scores.
Brooks et al., 2010	Exploratory study (a compilation of results of three studies)	within subject	114	Driving simulator (the body of a car and three screens)	2-min training session, four 5-min trials	Motion Sickness Assessment Questionnaire	MSAQ – before the study, prior and after each session = 11 times in total	For some participants the MSAQ scores increased at first, but then decreased as they adapted to the VR environment.

	Confirmation study		73		Three training sessions, three 30-min trials		MSAQ – before the study, prior and after each session = 9 times in total	
Newman et al., 2013		within subject	9	Flight simulator with a motion base and a cockpit	6 immerisions (on days 1-5 and 22)	Assessing the symptoms on a 0-10 scale (no symptoms – vomiting)	16 measures during a single trial	Rapid decrease after the first immersion, the effects do not wear off in time.
						Simulator Sickness Questionnaire	Before and after each trial	total score, nausea and disorientation scores decrease in time: total and nausea – Day 1-Day 4, Day 1-Day 5, effect retained for the last measurement; disorientation – Day 1-Day4.
Helland et al., 2016		within subject	20	Driving simulator (the body of a car and three screens)	Three 1-h trials, at least 2 days between the separate trial	One question: *"To what extent did you experience simulator sickness during the driving test?"*, rating on a 0-10 scale	After each driving trial = 3 times in total	The simulator sickness severity decreases with repeated simulator sessions, but the result was not statistically significant.
Reinhard et al., 2017		within subject	28	Two days, 7-14 days of break	Two days, 7-14 days of break. First day: 6 20-min immersions. Second days: 4 20-min immersions.	Fast Motion Sickness Scale	Once a minute	During both sessions the symptoms severity increases, less during the second session.
						Simulator Sickness Questionnaire	Before the immersions, after each immersion, at the end of the day	
