# Supplementary material for: Can Simulator Sickness Be Avoided? A Review on Temporal Aspects of Simulator Sickness
Source: Front Psychol. 2018 Nov 6;9:2132. doi: 10.3389/fpsyg.2018.02132 (PMC6232264; doi:10.3389/fpsyg.2018.02132)
Supplement: TABLE S3 — Studies focusing on how long the simulator sickness persists after VR exposure. [file Data_Sheet_4.PDF]

How long does the simulator sickness persist after VR exposure?

Authors and publication date	Study design (between or within subject)	Number of participants	Type of virtual reality technology used	Time of simulator/VR exposure	Method of measurement	Periods when simulator sickness was measured	Results (concerning the temporal aspect)
Tanaka and Takagi, 2004	within subject	14	HMD	Not given	Simulator Sickness Questionnaire	After exposure	The symptoms persist after leaving VR, the length of persistence depends on the initial severity of the symptoms: more severe – > 30 min, less severe – no longer than 5 min
Bos, MacKinnon and Patterson, 2005	within subject	24	Ship motion simulator – a ship bridge, motion platform, screen	Three sessions with no more than 1 day break, five 5-min tasks	Misery Scale (MISC – 0-10 scale)	Before the session, 8 times during the tasks (before, after task explanation, after each of the tasks + 1 additional); after leaving the simulator: after 2, 5, 10, 15, 30, 60, 90 and 120 min	Symptoms persist for some time, return to baseline in an hour after exposure. Only 4/24 subjects did not fully recover after 2 hours.
Keshavarz et al., 2018	between subject	121	Car simulator – body of a car, projection screen	Practice drive + four drives, each 5-7 min	Fast Motion Sickness Scale	Every minute during the task and after the exposure: immediately after and 1, 2 3, 5, 10 and 15 minutes after exposure	The participants who dropped out of the experiment needed more time to recover than those who finished it. Only 5 subjects did not fully recover after 15 min. All subjects – significant decrease of symptoms between immediately after exposure and 3 min later
Singer, Ehrlich and Allen, 1998	within subject	32	Virtual Research Corporation VR4 HMD	Not given	Simulator Sickness Questionnaire	Before VR exposure, "Mid-Experiment", "Post-Experiment", after 30 min rest	All of the symptoms, except for disorientation, return to baseline after 30 min.
McCauley et al., 1990	within subject	48	Flight simulator – motion system, a cockpit, screen	10 min training + 4 10-min trials	"SSEQ" – Simulator Sickness Questionnaire and a 8-point scale	Immediately after VR exposure and 30 min later	Severity of symptoms decreases between the measurement time points.
Braithwaite and Braithwaite, 1990	between subject	115	Helicopter simulator – with the body of a helicopter and screens	Not specified in detail, different across participants	After a session	Qualitative assessment	Six participants: severe headaches (lasting for 2-6 hours). Two participants: nausea (up to 2 hours post exposure). Six participants: other symptoms, e.g. visual flashbacks, unsteadiness or symptoms different than ones experienced during the VR exposure.

Moss, Muth, 2011	between subject	80	HMD; locating objects in VR (head movements)	Five 2-min trials	Simulator Sickness Questionnaire (orally; pre-recorded cassette)	SSQ: before the experiment (pre-practice), after the practice sessions, after each trial (5 times), 5 min post exposure, 10 min post exposure<br>= 9 times in total	10 min post exposure – significantly higher SSQ total score than the pre-practice score.
Moss et al., 2011	within subject	22	HMD	Two sessions separated by 7 days: 2 48-s practice trials + 5 2-min trials = circa 12 min each time	Simulator Sickness Questionnaire	Baseline, after practice trials, after each trial, 5 and 10 min post exposure	Symptoms return to baseline after a 10-min rest.
Biernacki, Dziuda, 2014<br>Dziuda et al., 2014	within subject	12	Truck simulator (a cabin of a truck + cylinder screen)	Two 30-min trials with one-week break (fixed-base and mobile platform)	Simulator Sickness Questionnaire	For each trial: before simulator exposure, 2 min after exposure, 30 min after exposure<br>= 6 times in total	Total SSQ score, nausea, disorientation and oculomotor disturbance levels higher 2 min after exposure than 30 min after exposure.<br>Total SSQ score, oculomotor disturbance and disorientation higher 2 min after exposure than prior to the experiment.
Malińska et al., 2014	within subject	20	Virtual workstation	Circa 2 hours, three sessions	Self-developed questionnaire (8 symptoms)	Questionnaire: straight after simulator exposure, 20 minutes later, up to 24 hours after simulator exposure (e-mail)<br>= 3 times in total	Simulator sickness symptoms reported 4 hours after simulator exposure - dizziness, headache, disorientation, drowsiness and 5 hours after 3D movie viewing - drowsiness, apathy, fatigue, eye ache, headache, dizziness, nausea (minor severity).
